# Cryptogenic organizing pneumonia associated with radiation: A report of two cases

**DOI:** 10.3892/ol.2013.1716

**Published:** 2013-11-29

**Authors:** SACHIKA NOGI, HIDETSUGU NAKAYAMA, YU TAJIMA, MITSURU OKUBO, RYUJI MIKAMI, SHINJI SUGAHARA, SOICHI AKATA, KOICHI TOKUUYE

**Affiliations:** Department of Radiology, Tokyo Medical University, Shinjuku, Tokyo 1600023, Japan

**Keywords:** cryptogenic organizing pneumonia, breast cancer, irradiation, non-small cell lung cancer

## Abstract

Cryptogenic organizing pneumonia (COP) following radiotherapy is occasionally diagnosed as radiation pneumonitis or bacterial pneumonia. The current study presents two cases of COP following radiotherapy: A 48-year-old premenopausal female with breast cancer and an 84-year-old male with non-small cell lung cancer. In the cases of breast cancer and lung cancer, patients were first diagnosed with bacterial pneumonia and radiation pneumonitis, respectively. In the two cases, computed tomography disclosed the migration of ground glass shadows, which were finally resolved without any fibrotic changes. The two cases were finally diagnosed as COP associated with radiotherapy. When an infiltrating shadow is present outside of the irradiated field, COP must be included in the differential diagnosis.

## Introduction

Lange initially described obliterative bronchiolitis in 1901 (1) and the unique clinical and histological characteristics of this pulmonary disease are well-known. Epler *et al* coined the term ‘bronchiolitis obliterans organizing pneumonia’ (BOOP) to describe this condition. In 1985, the authors described 50 patients with BOOP and showed that it was a clinicopathological entity that was distinct from irreversible pulmonary fibrosis (2). Histologically, BOOP is characterized by granulation tissue and polypoid masses in the lumens of the small airways, alveolar ducts and certain alveoli. Radiographs show an unusual pattern of patchy densities with a ‘ground glass’ appearance. In 2001, the American Thoracic Society and the European Respiratory Society standardized the classification of idiopathic interstitial pneumonia with the presence of BOOP as cryptogenic organizing pneumonia (COP) (3).

Idiopathic interstitial pneumonia may be associated with post-respiratory infection, drug addiction, connective tissue disease, organ transplantation, inhalation of fumes and radiotherapy (4,5). Crestani *et al* defined COP associated with radiotherapy for breast cancer as the presence of the following conditions: i) radiation therapy to the breast within 12 months of symptom onset; ii) general and/or respiratory symptoms lasting for ≥2 weeks; iii) infiltrations outside the irradiated volume; and iv) no other specific cause of the symptoms (6). Although there are a number of previous studies that have reported COP following radiotherapy for breast cancer, reports of COP following radiotherapy for non-small cell lung cancer (NSCLC) are rare. COP associated with radiotherapy is occasionally diagnosed as radiation pneumonitis or bacterial pneumonia. The current study presents two cases of COP following radiotherapy, one breast cancer patient and the other a patient with NSCLC. Written informed consent was obtained from the patients.

## Case reports

### Breast cancer case

**Figure 1:**
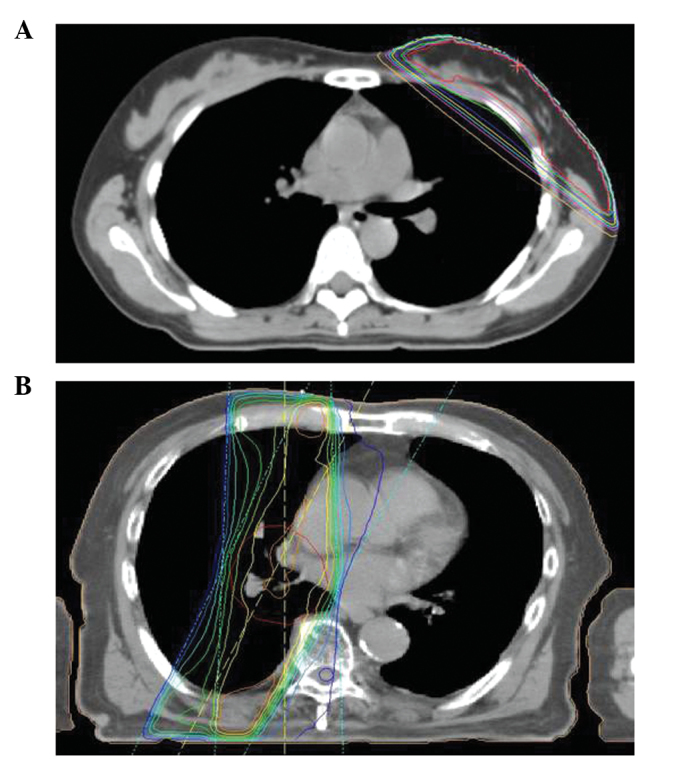
Dosimetry of (A) a 46-year-old female with breast cancer and (B) an 84-year-old male with non-small cell lung cancer.

A 48-year-old premenopausal female with cancer in the left breast underwent breast conservative surgery and sentinel lymph node biopsy. The pathological examination led to a diagnosis of stage I (pT1cN0M0) invasive ductal carcinoma that was estrogen receptor-positive and Her2 receptor-positive. The patient underwent a second surgery following observations of a positive surgical margin, and, three months following the initial surgery, underwent radiotherapy of 50 Gy in 25 fractions to the left whole breast using a 4-MV X-ray and a tangential field technique. An additional dose of 10 Gy in five fractions to the tumor bed was used as a boost therapy with 9 MeV electrons (Fig. 1A). The patient was treated with anti-estrogen therapy consisting of oral tamoxifen plus subcutaneous luteinizing hormone-releasing hormone agonist.

**Figure 2:**
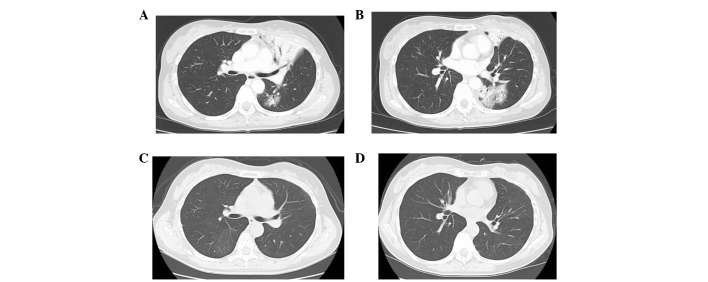
Chest computed tomography of the breast cancer patient following completion of radiotherapy showed (A and B) consolidation with air bronchograms in the left upper lobe and the superior segment of the left lower lobe at 4 months and (C and D) complete resolution in the left upper and left lower lobes at 13 months.

The patient experienced a fever (38.8°C) and developed a productive cough four months following the completion of radiotherapy. The patient’s laboratory results are shown in Table I. A chest X-ray showed an infiltrating shadow in the upper lobe of the left lung. Computed tomography (CT) showed consolidation with air bronchogram in the left upper lobe and ground glass densities in the left lower and right upper lobes (Fig. 2A and 2B). As these shadows appeared outside of the irradiated volume, the patient was diagnosed with bacterial pneumonia, despite no pathological confirmation. The patient was treated with oral levofloxacin (500 mg for 14 days). Four weeks following the completion of levofloxacin therapy, a CT scan showed that the original consolidation and ground glass densities had resolved, but new ground glass densities had appeared in the right upper lobe. Transbronchial lung biopsy revealed alveolar septal and focal intra-alveolar fibrosis; these observations were consistent with an observation of organizing bronchopneumonia. Examination of bronchoalveolar lavage (BAL) fluid showed the following: 34% lymphocytes, 5% neutrophils, 19% eosinophils and 42% macrophages. The CD4/CD8 ratio was 0.78. Based on these results, the patient was diagnosed with COP. After 8 months, all symptoms were spontaneously resolved (Fig. 2C and 2D). A CT showed only slight fibrosis in the lung that was consistent with the irradiated volume. The patient began taking herbal medicine containing *Scutellaria* root to treat menopause symptoms. However, two weeks following this, the patient experienced symptoms similar to those experienced with COP. The CT scan showed new ground glass densities in the right upper lobe and the patient was suspected to have a recurrence of COP. The patient stopped taking the herbal medicine, and symptoms and shadows on the CT resolved.

### NSCLC case

**Figure 3:**
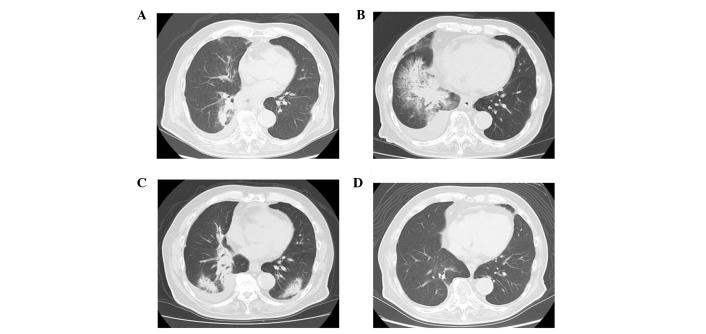
Chest computed tomography of the lung cancer patient following the completion of radiotherapy showed (A) consolidation with ground grass shadow in the irradiated field in the right lower lobe and right pleural effusion at 4 months, (B) consolidation of the extended irradiated volume and increased pleural effusion at 7 months, (C) constriction of the ground grass shadow and a migrated shadow in the lower two lobes at 11 months and (D) resolution in the lower lobe (i.e. no fibrosis) at 18 months.

An 84-year-old male exhibited clinical stage IIA (cT1bN1M0) squamous cell lung cancer in the right upper lobe and was considered unsuitable for surgery due to poor pulmonary function. The patient underwent radiotherapy to the primary tumor and metastatic lymph node, consisting of 60 Gy in 30 fractions using 10-MV X-rays (Fig. 1B). The patient was referred to Tokyo Medical University (Tokyo, Japan) due to a non-productive cough four months following the completion of radiotherapy. The patient was afebrile, but blood CRP levels were elevated (11.8 mg/dl). The patient’s laboratory results are shown in Table I. The chest radiograph showed a patchy shadow in the right middle lobe. CT disclosed consolidation in the right lower lobe that was consistent with the irradiation volume (Fig. 3A). The patient was diagnosed with radiation pneumonitis and prescribed oral prednisone at a dose of 30 mg per day. After two weeks, the patient’s symptoms and laboratory results improved, and the patchy shadow on the chest radiograph disappeared. The prednisone dose was tapered and discontinued after two months. The patient again developed a non-productive cough without fever three months following the first presentation with a cough. CT showed an extended consolidated shadow outside the irradiated volume (Fig. 3B) that was resolved following the administration of oral prednisone. The prednisone was discontinued and 11 months following the completion of radiotherapy, a follow-up CT showed an infiltrated shadow in the lower lobes (Fig. 3C). The white blood cell count was 4.8×10^3^ cells/μl and the C-reactive protein levels were 1.1 mg/dl. The patient was diagnosed with COP due to their responsiveness to prednisone, migration of the consolidation on CT and resolution of fibrosis following shadow consolidation. The patient again received daily oral prednisone (20 mg/day) for four months, which was tapered over seven months. The infiltrated shadows disappeared following 18 months of radiotherapy (Fig. 3D).

## Discussion

The two cases presented in the current study exhibited CTs that showed consolidations with a ground glass appearance. In the two cases, the shadows migrated and were finally resolved without any fibrotic changes in the CT examination. No observations of bacterial or viral infection were identified. These observations were compatible with COP according to the criteria of Crestani *et al*(6), who defined COP to be associated with radiotherapy.

Takigawa *et al* reported that four out of 157 (2.5%) patients who underwent radiotherapy following breast conservative surgery developed COP (7). The use of tamoxifen, chemotherapy and breast position were factors that were not associated with developing COP. However, the eosinophil and neutrophil counts were increased in the BAL fluid and the CD4/CD8 ratio was >2.0. In the two present cases, the eosinophil count was elevated but the CD4/CD8 ratio was <1.0. Toma *et al* previously reported that there were increased mast cells in the BAL fluid of five patients who had developed COP following radiotherapy (8). In addition, Roberts *et al* reported that the CD4/CD8 ratio in the BAL fluid was elevated in one patient with COP following radiotherapy (9), while Nagai *et al* found that a decreased CD4/CD8 ratio was consistent with idiopathic COP (10). Overall, these disparate observations suggested that the CD4/CD8 ratio in the BAL fluid of patients with COP associated with radiotherapy is uncertain, as detailed subset examinations of lymphocytes in BAL fluid have not been previously conducted.

Previously, Ogo *et al* conducted a multi-institutional survey that found that 37 out of 2,056 (1.8%) patients with breast cancer exhibited COP (11). However, the authors did not identify any correlation between patient characteristics and the occurrence of COP. Kubo *et al* reported that 12 out of 413 (2.9%) patients exhibited COP (12). The central lung distance (>1.8 cm) was the only significant risk factor; notably, nodal status, location, field size, use of chemotherapy and ipsilateral V20 were not risk factors. Katayama *et al* reported that 16 out of 702 (2.3%) patients developed COP following breast conservative surgery (13). In that study, multivariate and univariate analyses showed that an age of >50 years and endocrine therapy were risk factors for COP. The authors noted that radiation-induced COP occurred >4 months following radiotherapy, consistent with the cases of the present study. These results suggested that the COP occurred in 2–3% of patients with tangential breast irradiation.

Murai *et al* previously reported that nine out of 189 patients developed COP following stereotactic radiotherapy of the lung (14) and calculated the COP incidence at 1 and 2 years to be 4.0 and 5.2%, respectively. No association was found between COP and radiation dose, but prior radiation pneumonitis was a marked risk factor for COP. The authors suggested that following stereotactic radiotherapy, patients must be carefully followed for ≥2 years, particularly those with symptoms consistent with radiation pneumonitis. To the best of our knowledge, COP following conventional irradiation of the lung has not been reported previously. In the current case, a consolidation shadow was identified in the right lower lobes below the irradiated volume. Previously, Oie *et al* observed that COP appeared near the area affected by radiation pneumonitis (15).

Numerous studies have previoulsy reported that a marked improvement may be achieved by the administration of corticosteroids, but relapses often occur when the dose is tapered. Katayama *et al* reported that two out of 16 patients required corticosteroids for >3 years (13), while Narabayashi *et al* described radiation-induced COP that was refractory to corticosteroids (16). Stover *et al* suggested that macrolides may be useful in these cases (17). By contrast, Ogo *et al* reported that clinical and radiology observations improved in 50% of patients without corticosteroid treatment (11), as was demonstrated in the current breast cancer case.

In conclusion, when an infiltrating shadow is present outside of the irradiated field, COP must be included in the differential diagnosis.

## Figures and Tables

**Table I:** Patient laboratory results.

Parameters	Breast cancer patient	Lung cancer patient
Hematology		
WBC count, cells/μl	9.9×10^3^	6.2×10^3^
Neutrophils, %	86.7	74.4
Lymphocytes, %	6.8	15.2
Eosinophils, %	2.4	2.4
RBC, cells/μl	4.55×10^6^	4.24×10^6^
Hb, g/dl	12.8	13.7
Ht, %	38.5	41.1
Plt, cells/μl	3.79×10^5^	2.11×10^5^
Serology		
CRP, mg/dl	9.5	11.8
KL-6, U/ml	310	833
Biochemistry		
TP, g/dl	7.3	7.0
T-Bil, mg/dl	0.35	0.97
GOT, IU/l	30	20
GPT, IU/l	39	24
LDH, IU/l	215	168
ALP, IU/l	750	274
γ-GTP, IU/l	177	54
BUN, mg/dl	8.5	19.6
Cr, mg/dl	0.54	0.66
Na, mEq/l	143	141
K, mEq/l	4.0	4.3
Cl, mEq/l	106	104
BAL fluid		
Total cell count, cells/ml	2.5×10^7^	-
Macrophages, %	42.4	-
Lymphocytes, %	34.0	-
Neutrophils, %	4.8	-
Eosinophils, %	18.8	-
CD4/CD8	0.78	-

WBC, white blood cell; BAL, bronchoalveolar lavage.
